# Observations on sex ratio and behavior of males in *Xyleborinus saxesenii* Ratzeburg (Scolytinae, Coleoptera)

**DOI:** 10.3897/zookeys.56.530

**Published:** 2010-09-17

**Authors:** Peter H.W. Biedermann

**Affiliations:** Department of Behavioural Ecology, Institute of Ecology and Evolution, University of Bern, Baltzerstrasse 6, 3012 Bern, Switzerlan

**Keywords:** inbreeding, haplodiploidy, ambrosia beetles, all male broods, LMC, subsociality

## Abstract

Strongly female-biased sex ratios are typical for the fungalfeeding haplodiploid Xyleborini (Scolytinae, Coleoptera), and are a result of inbreeding and local mate competition (LMC). These ambrosia beetles are hardly ever found outside of trees, and thus male frequency and behavior have not been addressed in any empirical studies to date. In fact, for most species the males remain undescribed. Data on sex ratios and male behavior could, however, provide important insights into the Xyleborini’s mating system and the evolution of inbreeding and LMC in general.

In this study, I used in vitro rearing methods to obtain the first observational data on sex ratio, male production, male and female dispersal, and mating behavior in a xyleborine ambrosia beetle. Females of Xyleborinus saxesenii Ratzeburg produced between 0 and 3 sons per brood, and the absence of males was relatively independent of the number of daughters to be fertilized and the maternal brood sex ratio. Both conformed to a strict LMC strategy with a relatively precise and constant number of males. If males were present they eclosed just before the first females dispersed, and stayed in the gallery until all female offspring had matured. They constantly wandered through the gallery system, presumably in search of unfertilized females, and attempted to mate with larvae, other males, and females of all ages. Copulations, however, only occurred with immature females. From galleries with males, nearly all females dispersed fertilized. Only a few left the natal gallery without being fertilized, and subsequently went on to produce large and solely male broods. If broods were male-less, dispersing females always failed to found new galleries.

## Introduction

In panmictic (randomly mating) species, natural selection usually favors balanced sex ratios (Fisher 1930). In settings where male and female offspring are of unequal value to the mother, however, optimal sex ratios may be unbalanced. For example, in a non-randomly mating population or species with strictly local offspring reproduction, a mother will gain highest fitness by producing as many daughters as possible and just enough sons to ensure fertilization of all sisters in the brood (Hamilton 1967). This extreme economy in the production of males is common in small arthropods with regular brother-sister mating and local mate competition (LMC) between brothers (Hamilton 1967; Norris 1993). Usually it is associated with arrhenotokous haplodiploidy (Hamilton 1978 referred to arthropods exhibiting these characters as “the biofacies of extreme sex ratios and arrhenotoky”). Arrhenotoky potentially gives a mother precise control over the sex of each offspring, as she may choose to produce diploid daughters by fertilizing an egg, or haploid sons by leaving it unfertilized (Hamilton 1967; Charnov 1982). Hamilton (1978) suggested that the ancestral habitat for the aforementioned biofacies is situated under the bark of dead trees, even if the support for this quotation is weak and claims on the original habitat of ancient lineages are highly speculative (Normark et al. 1999).

A prominent group of beetles living under the bark is the weevil sub-family Scolytinae, that includes species with varying mating systems (Kirkendall 1983; 1993) and ploidy (diploid, functional haploid, haplodiploid; Kirkendall 1993; Brun et al. 1995; Normark et al. 1999). Both characters are selected to the extremes in all species belonging to the subtribe Xyleborini, which are a typical example for Hamilton’s biofacies since they are characterized by strong inbreeding, LMC and haplodiploidy. These so-called ambrosia beetles solely feed on a microbial complex (fungi, yeasts and bacteria; Haanstad and Norris 1985), which they grow on the walls of self-constructed tunnel systems (“galleries”) in the heart- or sapwood of trees. Until recently, this habitat has virtually been impossible to access and observe without destruction, which is why basic data on life histories, mating systems and behavior of most Xyleborini is still missing. The adaptation of an in vitro rearing technique (Norris and Baker 1967; Biedermann et al. 2009) finally may lead to the establishment of an excellent new model system for studying the independent evolution of inbreeding and haplodiploidy in a weevil lineage.

The few observational studies on Xyleborini that exist suggest that these beetles behave subsocially (Kirkendall et al. 1997; Mueller et al. 2005). Subsociality in Xyleborinus saxesenii Ratzeburg and other Xyleborini is indicated by the fact that mature female offspring maintain the natal gallery throughout a few days or even weeks at a time when they could already found their own nests (Kirkendall et al. 1997; Mueller et al. 2005; Biedermann 2007). Costs of independent breeding have been found to be high in this species due to risky dispersal and low founding success, which in former studies did not exceed 20% neither in the field nor when in vitro culturing was used (Biedermann et al. 2009). Therefore, it might pay for daughters to stay in productive galleries, where they either reproduce themselves, or gain indirect fitness benefits by helping to produce more sisters (e.g. Peer and Taborsky 2007). The help of males (if there is) is assumed to be of minor importance to the family, given their tiny size (figures in Fischer 1954) and their underrepresentation in relation to females.

If males do not play an important role for gallery maintenance and protection, male numbers should be minimized in order to lower the cost of LMC. The optimal number should be affected by the maximum number of females one male is able to fertilize, the timing of male production, male survivorship and longevity, as well as male dispersal (see Kirkendall 1993 for a detailed review; Borsa and Kjellberg 1996). In Xyleborini males are outnumbered by females by a ratio of 1:5 to 1:30, depending on the species (e.g. Schedl 1962). Males supposedly hatch before females (observed in Xyleborus affinis Eichhoff; Roeper et al. 1980), but their life expectancy is unknown. As they lack functional flight wings, they are assumed to never leave the nest (Roeper et al. 1980; Kirkendall 1983; 1993), however, recent data suggests that males of Xylosandrus germanus Reiter sometimes leave their natal gallery to crawl on the host tree in search for outbreeding opportunities, i.e., disperse (Peer and Taborsky 2004).

The objective of this study was to observe males of Xyleborus saxesenii inside their galleries for the first time to report their number and hatching time relative to females, as well as to determine whether or not males disperse and if they share in gallery maintenance and protection. Furthermore, I aimed at clarifying the existence of all male broods, which has been remarked upon in several studies on Xyleborini (e.g. Fischer 1954; Norris and Baker 1967; Kirkendall 1993). Such broods are assumed to be produced by unfertilized females that later mate with one of their sons to subsequently produce “normal” mixed broods (Herfs 1959). Their existence was tested by following the fate of unfertilized females. All these studies were conducted by consecutively rearing several generations of Xyleborus saxesenii families in an artificial medium in glass tubes that allow for behavioral observations (Biedermann et al. 2009).

## Methods

### Study species

In the study species Xyleborinus saxesenii Ratzeburg, the ambrosial complex is typically dominated by the anamorphic fungus Ambrosiella sulfurea Batra (Batra 1967), and serves as the sole food for adults as well as the developing larvae. The latter feed xylomycetophagously (on fungus and wood; Schedl 1958), in this way digging out a single large brood chamber where individuals of all age classes live in close vicinity to each other and also to their fungal food source. Galleries are always founded by individual females that usually have been fertilized by a brother prior to their emergence from the natal nest. The first offspring mostly stay with their mother after reaching adulthood while the subsequent offspring generations develop (e.g. Kalshoven 1962; Schedl 1966; Bischoff 2004; Biedermann et al. 2009). Brood care and fungus tending in Xyleborinus saxesenii are hitherto unknown, but expected to occur, because in case the foundress dies before the first brood has eclosed, the brood dies as well and the fungal garden degrades (Batra and Michie 1963; Norris 1979; 1993). Furthermore, gallery protection by blocking the entrance with the abdomen or a plug of frass and brood care behaviors have been observed in adult daughters of this as well as other xyleborine species (Kirkendall et al. 1997; Bischoff 2004; Biedermann 2007; personal observations).

### Laboratory breeding and data collection

Xyleborinus saxesenii beetles were bred in artificial agar-sawdust based “standard medium” in glass tubes (Biedermann et al. 2009). Single females were surface sterilized by submerging them first in ethanol (95%) and then in distilled water for a few seconds and subsequently put directly onto the medium. They usually started to excavate a tunnel system within two days and would not lay eggs until their mutualistic ambrosia fungus had started to grow. About 20% of the females started to lay eggs, which resembles the breeding success found in the field (Biedermann et al. 2009). The developing brood would subsequently enlarge the gallery system which was often constructed next to the tube wall, which facilitated the observations necessary for this study. Parts of the gallery inside the medium could not be accessed with this method, but as individuals move a lot, this should not have influenced my results.

### Observations

**Figure 1.**
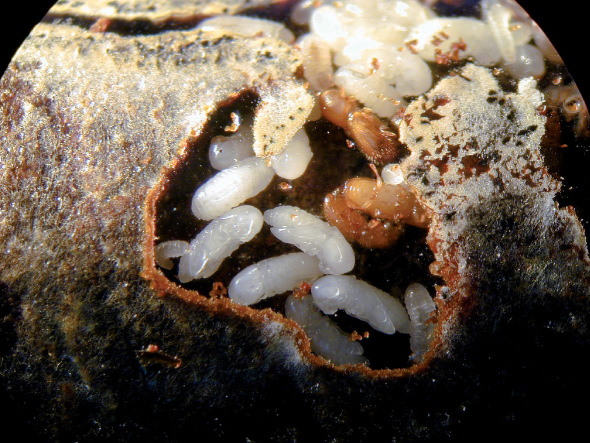
Parts of a brood chamber of Xyleborinus saxesenii in artificial medium. 1st and 2nd/3rd instar larvae, several pupae, two immature females and a male are visible. The male is mounting an immature female.

I scanned the beetles in 70 observable galleries every second to third day and recorded male and female numbers, allocating the individuals to different developmental stages (eggs, larvae, male and female pupae, immature females, mature females, and males). Male and female pupae and adults were easy to differentiate because of their strong sexual dimorphism in size (Fig. 1, see figures in Fischer 1954). Immature females were identified by their light brown coloration that would turn black after maturation. As a result of sex specific mortality rates and siblicide, the so gained “secondary sex ratio” of the immature and mature offspring may differ from the mother’s optimal “primary sex ratio” of the eggs. Where I could witness male-female interactions, I recorded the age of the partners and whether it was a mating attempt or successful mating. Whenever possible, I determined the dates when the first egg was present (*N* = 70 galleries), when the first male and female offspring hatched (*N* = 29 galleries), when the first and last female maturated (*N* = 26), and when the first male dispersed (*N* = 13). Dispersal was defined as emergence from the gallery, i.e., when individuals were found on the surface of the medium under the cap of the tube (Biedermann 2007). I stopped monitoring and dissected the galleries either when eclosion of new beetles ceased within about 3 months (*N* = 41 galleries), or when all adult females had dispersed (*N* = 29 galleries). In the latter case, galleries were used to monitor male dispersal.

I measured the breeding success (defined as the successful start of egg-laying within 40 days) and brood size of 311 daughters out of 33 families (*median* = 5 daughters/family, *range* = 1–41) to determine whether they differ between daughters of galleries with males and male-less ones. Subsequently, I correlated the secondary sex ratio of 62 daughter-families that had successfully produced a brood with the secondary sex ratio of their mothers’ families to test whether secondary sex ratios might be heritable.

Additionally, I observed the behavior of eight males from different galleries for 10 min and calculated the proportion of time spent on different behaviors. I differentiated between shuffling (moving frass and feces with the legs under the body and with the abdomen), digging (excavating new tunnels), cannibalism (feeding on a larva, pupa, or adult beetle), courtship behavior (grooming an egg, larva, pupa, or adult beetle with maxillae and labium), walking, cropping (feeding on the fungal layer covering gallery walls), and mating attempt (mounting or copulating with a female) (Biedermann 2007).

### Statistical analyses

The association between numbers of male and female offspring was explored using linear regression. The number of males within a brood of a given size should have a binomial distribution under random sex determination. To test if this was the case, I compared the variance of the male numbers observed with that expected if the numbers of males were binomially distributed using a *Chi²*-test (Green et al. 1982). The same test was also used to analyze whether the number of males per family resembled a Poisson distribution. Male mating preference with either immature or adult females was analyzed using Fisher’s exact test. Usually the data were not normally distributed (which was determined with Kolmogorov-Smirnov tests), consequently non-parametric statistics were conducted. The Mann-Whitney *U*-test for comparisons between two independent groups was applied to determine productivity differences between male and male-less broods, and to check for differences in the timing of male and female hatching and dispersal. The inheritance of sex ratios between mother and daughter galleries was analyzed with a Spearman’s rank correlation. Analyses were performed with SPSS (Release 14.0; SPSS, Chicago, IL) and R (R Development Core Team 2008).

## Results

### Sex ratio and breeding success

**Figure 2.**
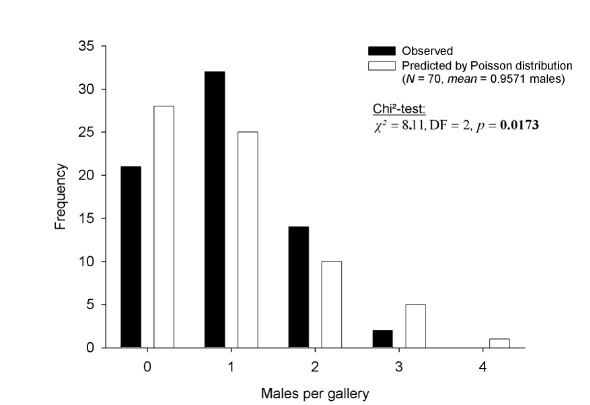
Occurrence of males in laboratory galleries of Xyleborinus saxesenii. The maximum number of males per gallery across the observation period is reported (N = 70 galleries). The data significantly differs from the expected Poisson distribution.

Males were extremely scarce in Xyleborinus saxesenii galleries (mean abundance per gallery = 0.96, SE = 0.09, *N* = 70; Fig. 2). Male production by mothers was not random, as their overall number did not resemble a Poisson distribution. They produced more one- and two-male broods, fewer male-less and three-male broods than expected, and no four-male brood, which suggests the optimal number is one or two males per brood. Nearly one third of the mothers produced no males (*N* = 21).

**Figure 3.**
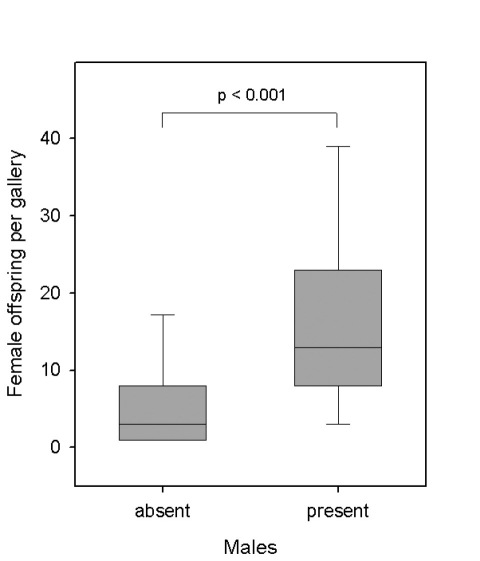
Presence of males in relation to the productivity of laboratory galleries of Xyleborinus saxesenii. Box-whisker plots with *median*, 90%, 75%, 25%, and 10% quartiles are shown. Mann-Whitney *U*-test: *T* = 423, *p* < 0.001, *N* = 21 + 49 galleries.

**Figure 4.**
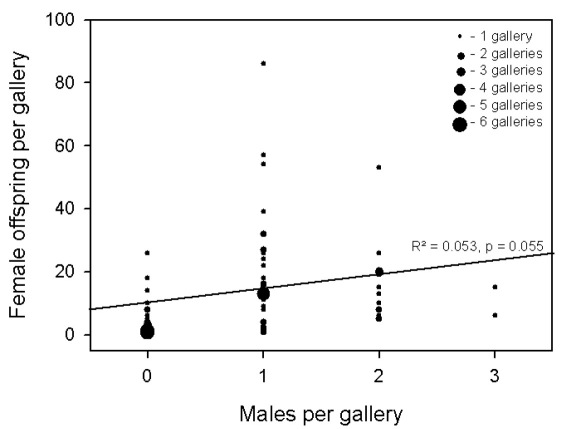
The number of males in relation to the number of females in laboratory galleries of Xyleborinus saxesenii. Each gallery is represented by one data point (N = 70). Linear regression (*males* = 0.785 + (0.0119 * *females*)): *F* = 3.824, *p* = 0.055, DF = 1.

Families with males were significantly larger than families without males (Fig. 3). In accordance with this finding, males were absent from 50% of the galleries with only ten daughters or fewer, whereas only 8.3% of the galleries with more than ten daughters were male-less. However, overall the number of males per gallery was only weakly affected by the number of adult females (Linear regression: *F* = 3.824, *p* = 0.055, DF= 1, *N* = 70; Fig. 4). The residual mean squared error of the number of males (MSE = 0.597) was much smaller than the mean variance expected under binomial distribution (0.9398; Chi²-test: *χ²* = 43.2, DF = 68, *p* > 0.05), which indicates that the production of males was not random, but relatively constant and precise (around one or two males per brood).

Breeding success (i.e., the likelihood to lay at least one egg within 40 days) of daughters per family ranged between 0% and 78% (*mean* = 15.2%, *median* = 0, *N* = 311 daughters from 33 families), and was 0% for all male-less broods. A statistical difference was however not detectable because of the high variation of breeding success across all families tested (*U*-test: *T* = 77, *p* = 0.067, *N* = 26 vs. 7).

The secondary sex ratios of the 62 successfully founded daughter-galleries did not correlate with the secondary sex ratios of their mothers galleries (Spearman’s rank correlation: *r* = 0.038, *p* = 0.771, *N* = 62).

### Male presence and behavior

**Figure 5.**
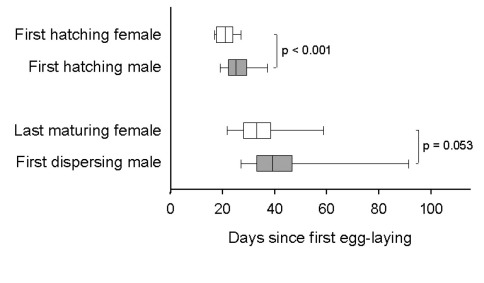
Timing of male and female hatching, maturation, and dispersal in laboratory galleries of Xyleborinus saxesenii. The correlation of the first male and female hatching from the pupal stage and the correlation between the last date a not-fully sclerotized female was seen, and the first male dispersal event (N = 29 galleries) were determined where possible and contributed one data point per gallery to this graph. Box-whisker plots with *median*, 90%, 75%, 25%, and 10% quartiles are shown. Mann-Whitney *U*-tests: *T* = 1066, *p* < 0.001, *N* = 29 + 29 galleries (female vs. male hatching); *T* = 325.5, *p* = 0.053, *N* = 26 + 13 galleries (female maturation vs. male dispersal).

**Figure 6.**
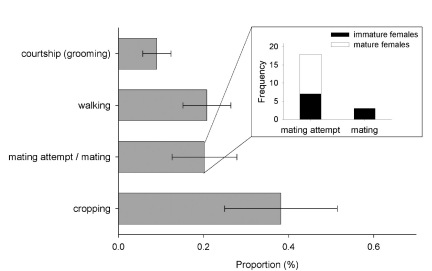
Behaviors of males in laboratory galleries of Xyleborinus saxesenii. Males (*N* = 8 galleries) were observed for 10 min and proportion of time spent with the different behaviors was calculated. The grey bars show the *mean* +/- SE for each behavior across all males. Male mating behaviors were further split into mating attempt and mating, and the age class of the female partner is reported.

The first male per gallery usually eclosed a few days after his first sister did so (Fig. 5), but before her maturation (*N* = 29 galleries). Following eclosion, males spent most of their time with cropping fungus and walking through the gallery system. When encountering a possible partner, the male started to courtship her, which resembles grooming the body of the female, and afterwards attempted to mate with her. Females seemed relatively reluctant, and thus mating attempts usually lasted several minutes and were rarely successful. Thirty mating attempts were observed, during which males mounted immature (*N* = 7 of 30 times) and mature females (*N* = 11 of 30 times) as well as pieces of wood, larvae, and other males (*N* = 12 of 30 times). Successful copulations, however, were only observed with three immature females, which hints towards a preference to mate with them (Fisher’s exact test: *p* = 0.09, *N* = 21; Fig. 6). Only two females were found to emerge unfertilized from their galleries (indicated by the production of an exclusively male brood later on); they emerged from galleries with brood sex ratios of 2:16 and 1:86, respectively, which indicates that one male is capable of fertilizing up to 60 sisters on its own (no unfertilized females were detected in a gallery with a sex ratio of 1:60). Other male behaviors which were rarely exhibited included digging, cannibalism, and the shuffling of saw-dust and feces with the legs (all with *median* = 0%).

Although males are wingless, some of them (at least one male in 13 out of 29 galleries) dispersed, i.e., were found on the surface of the medium, when all offspring had matured and no new eggs were laid. Males did not disperse randomly together with their sisters throughout gallery life, but in most cases the first male dispersal occurred after the last female (in a gallery) had matured (Fig. 5).

## Discussion

Under inbreeding conditions, natural selection should favor the production of that number of males that maximizes the mean number of inseminated females dispersing from a brood. Thus, in case males are able to inseminate only a limited number of females, the numbers of males and females should be correlated (e.g. Green et al. 1982; Borsa and Kjellberg 1996). In contrast to these predictions, male numbers in Xyleborinus saxesenii families were relatively independent of the number of sisters to be fertilized, ranging from 0 to 3 males per 1 to 86 females, and also independent of maternal brood sex ratios. These facts conform to a strict LMC strategy (e.g. Hamilton 1967; Bernal et al. 2001) with a relatively precise and constant number of males per brood. Males were apparently extremely successful to locate all their unfertilized sister. Only ~3% (2 of 70 galleries) of the successfully breeding females turned out to be unfertilized (they produced solely male broods), which is approximately the same ratio found for Xyleborus compactus Eichhoff in the field (Brader 1964). Additionally, in a family with a sex ratio of 1:60 no unfertilized females were found during consecutive lab rearing. The success of males is probably explainable by the gallery morphology. Xyleborini in the genus Xyleborinus usually construct brood chambers (“caves”; Wood 1982; Roeper 1995) instead of branching tunnel systems as they are excavated by species of the genus Xyleborus (J. Hulcr personal communication), where males and females probably meet more regularly and thus fewer males may be sufficient to fertilize all sisters. In line with this idea, male and female numbers are correlated in the branching tunnel systems of Xyleborus affinis Eichhoff (Xyleborina, Scolytinae), as founder females lay eggs in clusters that always contain a male egg (Roeper et al. 1980).

At least one male per family should, however, be expected under the assumption of strict inbreeding. Contrary to that, 30% of all families were male-less; a proportion which is not untypical for Xyleborini (19% of all families in Xylosandrus germanus Reiter in the field; Peer and Taborsky 2004). As these measures were taken on adult offspring, the easiest explanation for that finding is that the secondary sex ratios reported, differ from the primary sex ratios allocated by the mother to the eggs, i.e., males had somehow been eliminated. Such secondary male killing could have been accomplished either by selectively cannibalizing offspring or by selecting mechanisms of the fungal cultivars (for attine ants; Mueller 2002) or of bacteria in the ovaries (Peleg and Norris 1972; Kawasaki et al. 2009). Given that both microbes are transmitted only from mothers to daughters, they would presumably profit from female biased broods (Dyson and Hurst 2004; Normark 2004). Studies on the primary sex ratio allocated by the mother to the eggs are therefore necessary to clarify the role of secondary sex ratio distorters as a cause for the high frequency of male-less families in Xyleborini. If, however, it is assumed that there is no secondary male killing and that the absence of males is intended by the mother beetle from the very beginning, then either the chances for mating with foreign partners (and thus, outbreeding) must be higher than previously expected, or selection against male-less broods must be weak. Meeting and outbreeding with foreign mates is obviously possible, as males dispersed from nearly half of the galleries when brood production ceased and females sometimes produced all male broods, whose males also all emerged. It fits these data that Xyleborinus saxesenii males can sometimes be seen outside their nests, occasionally walking on their natal host trees (personal field data). The idea that males disperse from their natal nest in search for outbreeding opportunities implies, however, that they are able to enter foreign galleries, what seems so be complicated by the fact that females or more rarely males (in the genera Coptodryas and Cyclorhipidion; J. Hulcr personal communication) block the gallery entrances (e.g. Kirkendall et al. 1997; personal observations). On the other hand, male-less families were always very small (equal to or less than 10 females; see similar results in Hypothenemus hampei Ferrari by Borsa and Kjellberg 1996), and in a previous study daughters of small families (less than 20 females) were found to contribute much less offspring to the next generation than the daughters from larger families, presumably because the latter are in better body condition and have a more productive complex of microbes to nourish them (Biedermann et al. 2009). Therefore, it is possible that these small male-less galleries would anyway not contribute much fitness to the next generation and hence selection against the absence of males within small broods is probably weak.

Relatively independent from the fact if secondary manipulations of the sex ratio occur, male numbers should be higher under conditions that allow outbreeding, as has been shown in Xyleborinus germanus (Peer and Taborsky 2004). In case Xyleborinus saxesenii mothers also allocate offspring sex according to outbreeding opportunities, then less males should have been found in my lab study compared to the field, where outbreeding opportunities can be expected to arise regularly. Contrary to these expectations, I found a sex ratio of 1:8 (m:f) on average, which is well below the average 1:20 sex ratio previously found in the field both in Australia (Hosking 1972) and Switzerland (personal observations). Although the methods for measuring sex ratios differed between lab and field studies, these strongly contradicting results are not explainable by methodological differences alone and may point to other (environmental) factors influencing sex allocation in Xyleborinus saxesenii.

Males hatched significantly later than their first sisters did, which contrasts with findings in Xyleborinus affinis, whose males hatch before females (Roeper et al. 1980). I hypothesize that in Xyleborinus saxesenii fertilization of all females by their brothers was ensured because females need about ten days to fully develop (Biedermann et al. 2009), and males hatched on average 4 days after females. Furthermore, fertilization of all females was made possible by the high sexual activity of males that did not engage in social behaviors. Instead, they constantly wandered the gallery in search for virgin females, which they started to courtship (groom) upon encounter, followed by mounting and copulation. Although rarely seen, these copulations occurred exclusively with immature females. Concordantly, males did not disperse as long as new immatures eclosed and could be fertilized, but tended to leave the natal nest as soon as eclosion ceased, presumably in search for outbreeding opportunities. This indicates that they try to fertilize as many sisters as possible, but search for outbreeding opportunities as soon as direct fitness maximization within the natal nest is no longer possible.

Unfertilized females produced solely male broods, but did not mate with their sons to subsequently produce “normal” mixed broods. This expectation was based on observations of Coccotrypes dactyliperda Fabricius (Scolytinae) females that do so (Herfs 1959). The behavioral difference between these two species is, however, plausible when one takes into account the extreme longevity and fertility of Coccotrypes dactyliperda females, which raise up to five broods, in this way producing up to 144 individuals in total. In contrast, Xyleborinus saxesenii females usually die soon after their first offspring matures (personal observations), which in case they mated with one of their sons would mean shortly after doing so. Even in case she was nevertheless able to lay a few fertilized eggs before her death, the brood would still be doomed as the mother’s presence and her brood care appears crucial for successful brood development (e.g. Norris 1993; Kirkendall et al. 1997; Biedermann 2007). Another fascinating explanation could be that male broods are intended by the females and represent an alternative reproductive tactic under certain conditions when fitness gains through outbreeding sons are higher than those that can be achieved with the production and maintenance of a mixed brood. Such a condition may, for example, be an environment with a lot of small and male-less broods (e.g., under high density), a situation in which selection would favor a few females to just produce sons that visit their male-less neighboring families.

My data shed light on the cryptic life and mating system of xyleborine ambrosia beetles within their galleries. In the future, molecular studies will be crucial for determining whether the observed inter- and intraspecific variance in sex ratios is an adaption to outbreeding in some Xyleborini or a hint for the existence of sex ratio distorters, for example, their symbionts. My data provide a starting point for future studies dealing with factors that influence xyleborine sex ratios and the frequency of outbreeding events. Ambrosia beetles are one of the best model systems for studying the evolution of inbreeding, haplodiploidy, sociality and symbioses, but at the same time one of the least known. Before comparative studies on different species can be done to address the issues mentioned, further basic data on the behaviors of these beetles inside their galleries have to be collected.

## Conclusion

My data suggest that males in Xyleborinus saxesenii Ratzeburg are extremely successful in locating and fertilizing all their sisters in the natal gallery. On average there is only about one male per family relatively independent of the number of sisters and the maternal brood sex ratio. Apparently, this is sufficient to fertilize all females, probably because the morphology of the gallery system with a central “brood chamber” makes it easy for males to locate them. Only about 3% of the females from broods with males disperse unfertilized from the natal nest and subsequently produce all male broods, but do not mate with one of their sons to produce a mixed brood afterwards.

Despite the fact that males are not the first to hatch within a brood, they do so before the first females mature, and only disperse from the natal gallery once the last female has finished her development. They do not engage in gallery maintenance, gallery protection and brood care, but constantly wander the gallery, presumably in search of unfertilized females. Although males attempt to mate with all individuals independent of age and sex, they were observed to copulate only with immature females.
